# Supplementary material for: Resistance to cancer immunotherapy mediated by apoptosis of tumor-infiltrating lymphocytes
Source: Nat Commun. 2017 Nov 10;8:1404. doi: 10.1038/s41467-017-00784-1 (PMC5680273; doi:10.1038/s41467-017-00784-1)
Supplement: Supplementary file 1 — Supplementary Information [file 41467_2017_784_MOESM1_ESM.pdf]

Description of Supplementary Files

File Name: Supplementary Information
Description: Supplementary Figures

File Name: Peer Review File

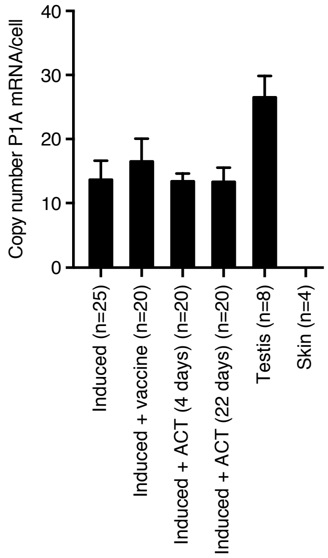

Supplementary Figure 1. Immunotherapy does not alter P1A expression in escaping tumors

Quantitative RT-PCR analysis of P1A expression was performed on total RNA extracted from cell suspensions obtained from homogenized induced TiRP tumors (500 mm^3) collected from mice that received a vaccine (or not) as indicated on Figure 1, or received ACT 4 days or 22 days before euthanasia. P1A mRNA levels were normalized based on β-actin expression and data are presented as copy number per cell. Testis and skin were used as positive and negative controls, respectively. Results are expressed as mean + s.e.m.

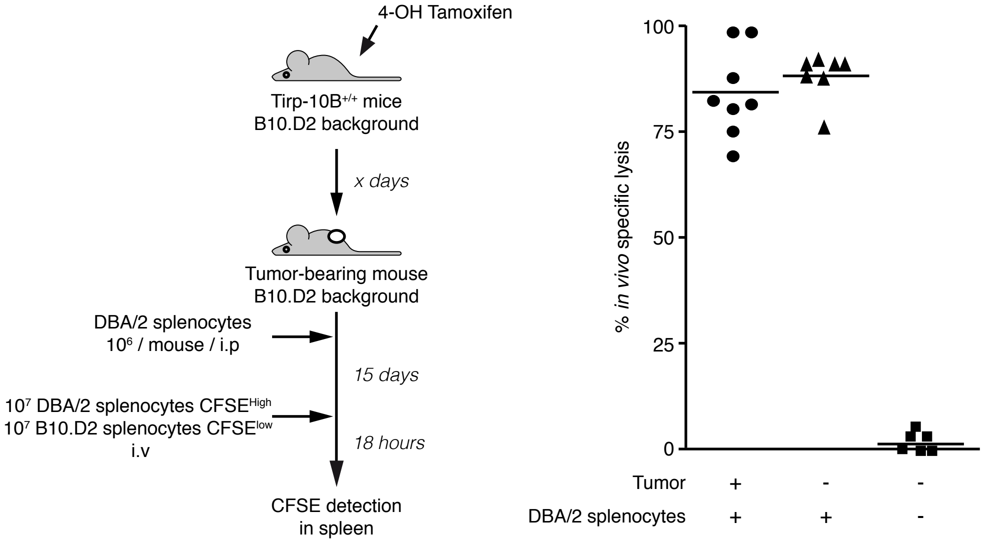

Supplementary Figure 2. Ability of tumor-bearing mice to mount an unrelated CTL immune response

Tumor-bearing TiRP mice (B10.D2 background) were immunized with splenocytes ($1x10^6$ *i.p.*) from DBA/2 mice, which have the same H-2 but differ for minor histocompatibility antigens (mHC). Two weeks later, an *in vivo* killing assay was performed to detect CTL responses against DBA/2 mHC antigens. Splenocytes from DBA/2 or TiRP-$10B^{-/-}$ (B10.D2;Ink4a/$Arf^{flox/flox}$) mice were labeled with 10 μM ($CFSE^{high}$) or 1 μM ($CFSE^{low}$) CFSE (Thermo Scientific), respectively, for 10 min at 37 °C in the dark. DBA/2 and B10.D2 splenocytes (10^7 each) labeled with different CFSE concentrations were injected into the immunized TiRP mice (*i.v.*). After 18h, the ratio between the two populations was determined in the spleen by FACS analysis. The percentage of specific lysis of DBA/2 targets was calculated based on this ratio (Tumor-bearing mice immunized: n=8; tumor-free mice immunized n=7; tumor-free mice not immunized n=6). Each dot represents an individual mouse, the mean value of each group is shown with a bar.

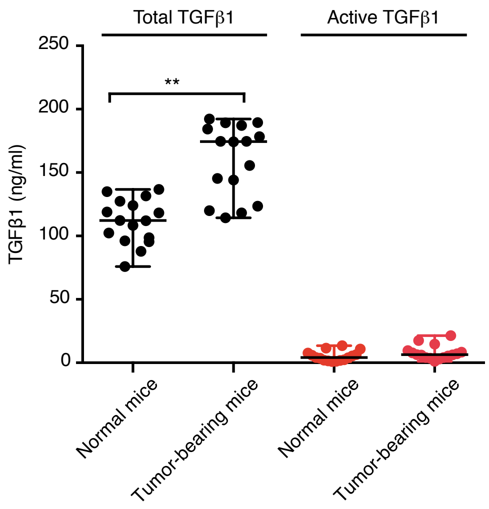

Supplementary Figure 3. TGFβ levels in the serum of mice bearing induced TiRP tumors.
Total (acid-activated) and active TGFβ1 levels were measured by ELISA in the serum of mice bearing induced Amela TiRP tumors or tumor-free mice. The amount of total and active TGFβ was evaluated by ELISA using mouse antibody to TGFβ1 (clone 21C11; a kind gift from Jacques Van Snick, Brussels). Briefly, a 96-well Maxisorb Immunoplate was coated overnight at 4°C with anti-mTGFβ1 antibody (5 μg/ml in 50 mM glycine buffer pH9). After saturation (1% BSA, 1h at 37°C) the samples were added and incubated for 2 h. Biotinylated anti-TGFβ1 antibody (clone 8D6H5, a kind gift from Jacques Van Snick, Brussels) was then added and incubated 30 min followed by the addition of Streptavidin-HRP. Ultra-TMB substrate was added and absorbance was acquired at 405 nm. For total TGFβ1, latent TGFβ1 was activated with acid prior to the ELISA by adding 20 μl of serum to 200 μl of DMEM+2% BSA, followed by addition of 10 μl of 4N HCl. The solution was then incubated for 1h, 4°C. The reaction was neutralized by the addition of 10 μl 4N NaOH. Unpaired t-test, two-tailed, **P<0.01. Each dot represents an individual mouse, median value with range is shown for each group.

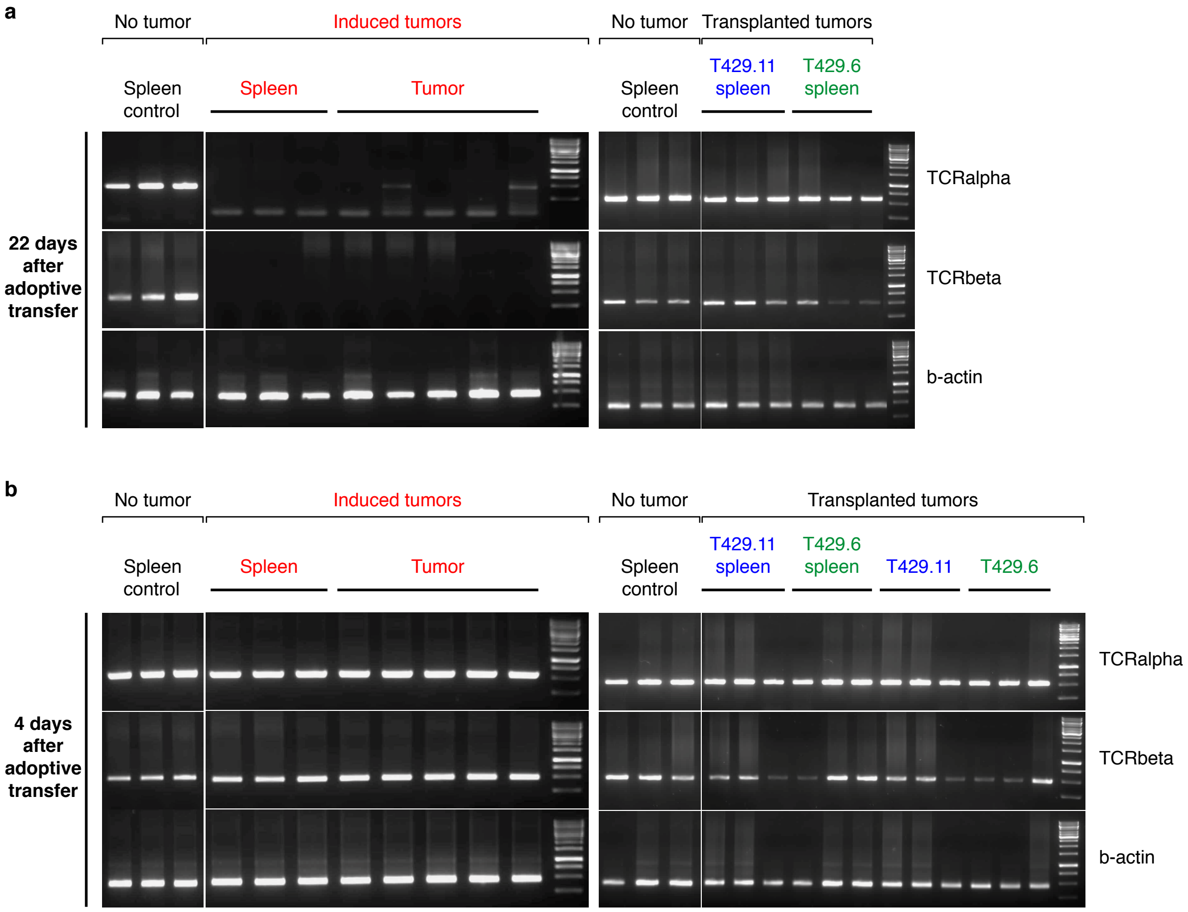

Supplementary Figure 4. Absence of TCRP1A $CD8^+$ cells in induced melanomas 22 days after ACT

(**a**) Mice bearing induced or transplanted tumors received ACT. After 22 days, spleen and induced tumors were collected and 100 ng of purified genomic DNA were subjected to TCR-clonotypic PCR employing primers specific for the CDR3 regions of the TCR alpha and beta chains of TCRP1A $CD8^+$ cells. Spleens from tumor-free mice that received ACT were used as controls. Transplanted tumors were fully rejected 22 days after ACT and therefore could not be tested. Genomic DNA (100 ng) isolated from frozen tissues was amplified by PCR using the following primers. TCRα chain: mVα8 forward 5'-GAA-TTC-ATG-CGT-CCT-GGC-ACC-TGC-3'; mJα10 reverse 5'-ACC-AGC-AAT-CGA-GTC-CCA-CTT-CCA-3'. TCRβ chain: mVβ1 forward 5'-GAA-TTC-ATG-AGC-TGC-AGG-CTT-CTC-3'; mJβ1.2 reverse 5'-AAA-AGC-CTG-GTC-CCC-GAC-CGA-AG-3'. β-actin: forward 5'-TGG-CGC-TTT-TGA-CTC-AGG-AT-3'; reverse 5'-AGC-CCT-GGC-TGC-CTC-AAC-3'.

(**b**) Same analysis as in (*a*) performed 4 days after ACT.

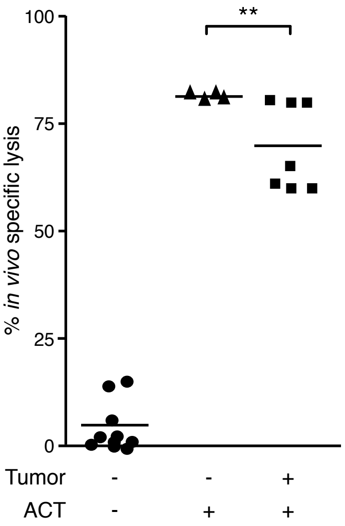

Supplementary Figure 5. ***In vivo*** **killing activity of TCRP1A CD8+ T cells in tumor-bearing mice after ACT**
Tumor-free mice and mice bearing induced TiRP tumors received ACT (*i.v.* injection of 10^7 activated TCRP1A CD8$^+$ T cells). Three days later, they received splenocytes (10^7 each, *i.v.*) pulsed with either peptide P1A$_{35\text{-}43}$ (LPYLGWLVF) or the irrelevant P91A$_{12\text{-}26}$ peptide (QNHRALDLVA), which were labeled with different concentrations of CFSE (10 μM (CFSE^{high}) or 1 μM (CFSE^{low}) CFSE, 10 min at 37 °C in the dark). After 18h, the ratio between the two populations was determined in the spleen by FACS analysis. The percent specific lysis of P1A-peptide pulsed splenocytes is indicated (Tumor-free mice without ACT: n=9; tumor-free mice with ACT: n=4; tumor-bearing mice with ACT: n=7). The reduced killing observed in tumor-bearing mice likely resulted from reduced numbers of TCRP1A CD8$^+$ T cells in the periphery due to intratumoral apoptosis. Unpaired t-test, two-tailed, **P<0.01. Each dot represents an individual mouse, the mean value of each group is shown with a bar.

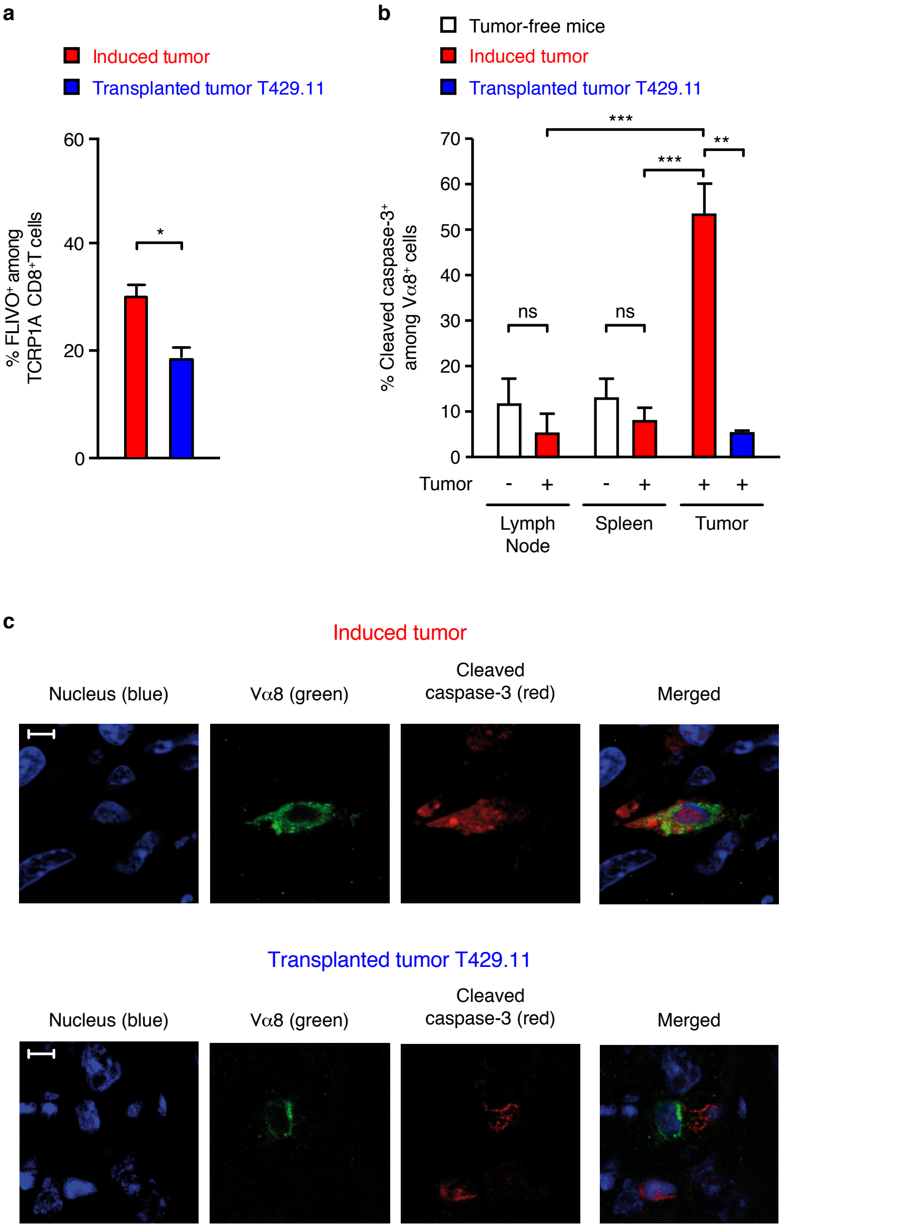

Supplementary Figure 6. Apoptosis of tumor-infiltrating TCRP1A CD8+ T cells
(**a**) Mice bearing induced or transplanted tumors were transferred with 10^7 activated TCRP1A CD8+ T cells pre-stained with CellTracker Blue CMAC Dye. Four days after transfer, mice received an *i.v.* injection of FLIVO (8.5 μg/mouse) four hours before euthanasia, as in Figure 3f. Apoptosis of TCRP1A CD8+ T cells was then evaluated on tumor cryosections (7 μm thick) using a MIRAX digital microscope and quantified using Biopix software. Mice receiving a non-targeting FLIVO control dye showed no staining of TCRP1A CD8+ T cells (n= 5 mice/group; 3 sections analyzed per mouse).
(**b**) Four days after ACT, draining lymph nodes, spleens and tumors from mice bearing induced or transplanted tumors were frozen and analyzed for apoptosis of TCRP1A CD8+ T cells, which express TCR Vα8[1]. Immunohistochemistry analysis was performed using anti-TCR-Vα8 antibody (KT50, Biolegend, 4 μg/mL) to detect TCRP1A CD8+ T cells and anti-cleaved caspase-3 antibodies (5AE1, Cell Signaling, 1:200) to detect apoptotic cells. The percentage of cleaved caspase-3+ cells among Vα8+ cells present in 6 random frames per tissue is shown. Lymph nodes from 4 tumor-free mice (277 Vα8+ cells) and 6 tumor-bearing mice (391 Vα8+ cells), spleens from 5 tumor-free mice (267 Vα8+ cells) and 7 tumor-bearing mice (299 Vα8+ cells), induced tumors from 18 mice (1211 Vα8+ cells), and transplanted tumors from 3 mice (221 Vα8+ cells) were analyzed by confocal microscopy.
(**c**) Illustrative image of induced (top) and transplanted (bottom) tumors collected 4 days after ACT, stained with anti-TCR-Vα8 (green) and anti-cleaved caspase-3 (red) antibodies, and analyzed by confocal microscopy. Scale bar = 5 μm. Results are expressed as mean + s.e.m. Unpaired t-test two-tailed (*a-b*), *$P<0.05$, **$P<0.01$, ***$P<0.001$.

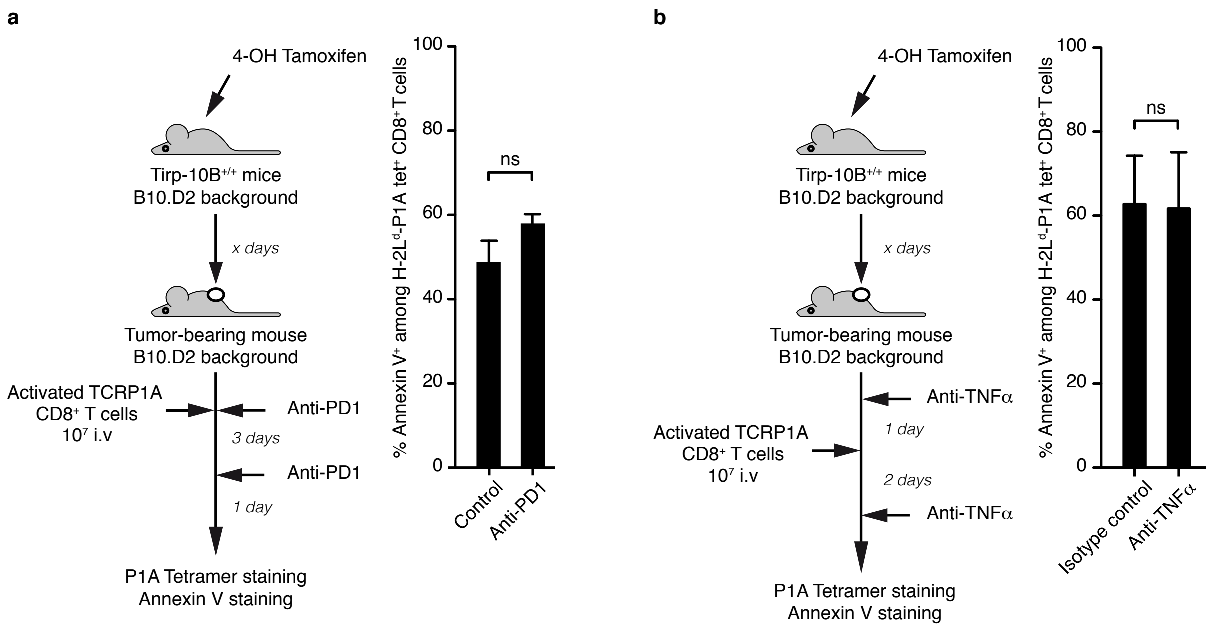

Supplementary Figure 7. No role for PD1 or TNFα in TCRP1A CD8$^+$ T-cell apoptosis
(**a**) Mice bearing induced TiRP tumors received (or not) 200 μg anti-PD1 antibody at the same time as ACT. A second injection was performed 3 days after ACT. One day after the second injection of anti-PD1 antibody, dissociated tumors were analyzed *ex vivo* by FACS for apoptosis of TCRP1A CD8$^+$ T cells (control: n=23; anti-PD1: n=3). (**b**) Mice bearing induced TiRP tumors received (or not) 100 μg anti-TNFα antibody (R&D System, AB410-NA) or isotype control (R&D System, A108-C) 1 day before ACT. A second injection was performed 2 days after ACT. Two days after the second injection of anti-TNFα antibody or isotype control, dissociated tumors were analyzed *ex vivo* by FACS for apoptosis of TCRP1A CD8$^+$ T cells (control: n=5; anti-TNFα: n=5). Results are expressed as mean + s.e.m. Unpaired t-test, two-tailed, ns: $P>0.05$.

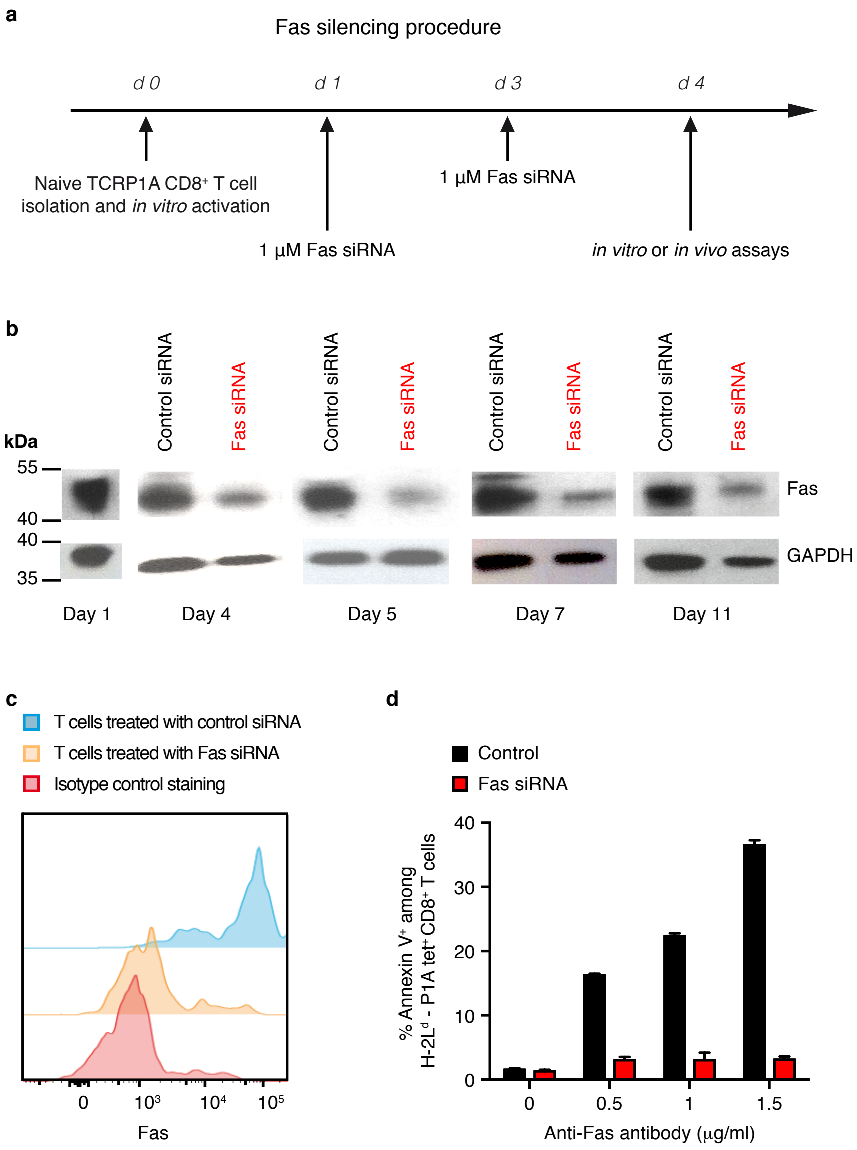

Supplementary Figure 8. Fas-silenced TCRP1A CD8+ T cells resist Fas-mediated apoptosis

(**a**) Protocol for Fas silencing showing the timing with regard to *in vitro/in vivo* use of the silenced TCRP1A CD8+ T cells. Naïve CD8+ T cells were isolated from TCRP1A transgenic mice and activated *in vitro* by co-culture with irradiated L1210.P1A.B7-1 cells on day 0. They were treated with siRNA (Accell) on days 1 and 3, and used for experiments starting on day 4.

(**b**) Activated TCRP1A CD8+ T cells were treated with control or Fas-specific siRNA and analyzed by Western blot at different time points using anti-Fas antibodies (Santa Cruz, sc-7886, 1:1000).

(**c**) FACS analysis of Fas surface expression 7 days after siRNA treatment of TCRP1A CD8+ T cells (Becton Dickinson, mAb Jo2).

(**d**) TCRP1A CD8+ T cells treated with control or Fas siRNA were incubated on day 4 with agonistic anti-Fas antibodies for 24 hours (Santa Cruz, sc-7886). Apoptosis was then measured by FACS using Annexin V. The results indicated that Fas-silenced TCRP1A CD8+ T cells resisted Fas-mediated apoptosis. Results are expressed as mean + s.e.m.

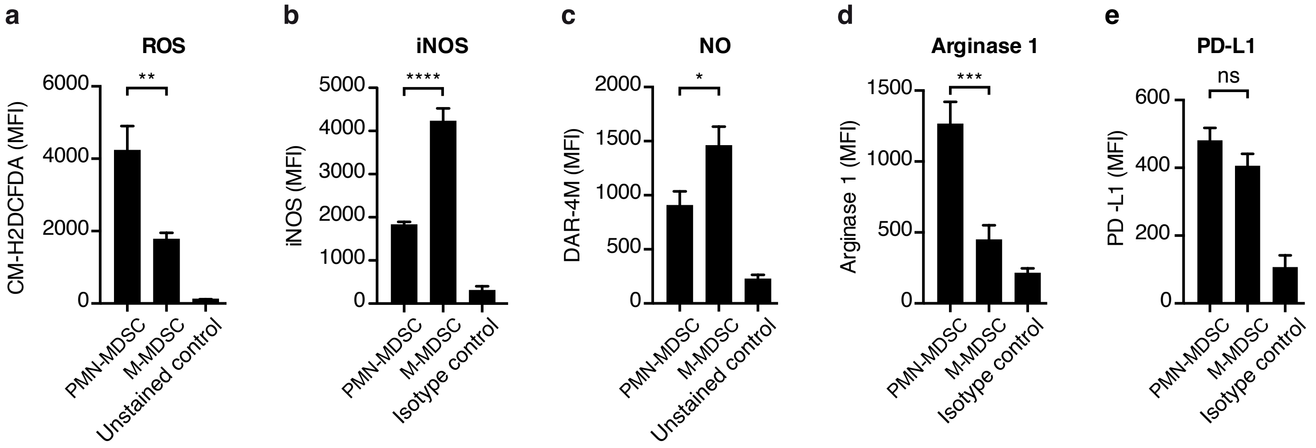

Supplementary Figure 9. Other molecules contributing to the immune-regulatory activity of MDSC subsets in Amela induced TIRP tumors.

Induced Amela tumors were homogenized and analyzed by FACS to distinguish the two different MDSC subsets (PMN-MDSC: Gr-1^{h}, CD11b^{+}, Ly6$C^{-/lo}$ Ly6G^{+} and M-MDSC: Gr-$1^{lo/int}$, CD11b^{+}, Ly6C^{h} and Ly6G^{-}). The levels of ROS (a), iNOS (b), Nitric Oxide (c), Arginase 1 (d) and PD-L1 (e) were evaluated by FACS and are shown as Mean Fluorescence Intensity (MFI) (n= 8).

To evaluate the production of Reactive Oxygen Species (ROS, a) and Nitric Oxide (NO, c), suspensions of tumor homogenates were incubated with 1 µM CM-H2DCFDA (Thermo Fisher; Cat: C6827) or with 1 µM DAR-4M (ENZO life sciences; ALX 620-069-M001), respectively, (dissolved in PBS) for 30min (37 °C). Cells were washed 3 times with PBS. The signal for ROS and NO was acquired by FACS together with MDSC markers. The expression of iNOS (b) and Arginase 1 (d) was evaluated by intracellular FACS staining using anti-Nos2 (Abcam; Cat: 15323, 1:100) and anti-Arginase 1 (R&D Systems; Cat: IC5868A, 1:20) antibody following the manufacturer's instructions (BD Biosciences; Cat: 554714). Expression of cell surface marker PD-L1 (e) was determined by FACS staining using an antibody to PD-L1 (Biolegend; Cat: 329714, 1:100). Results are expressed as mean + s.e.m. Unpaired t-test, two-tailed, *P<0.05, **P<0.01, ***P<0.001.

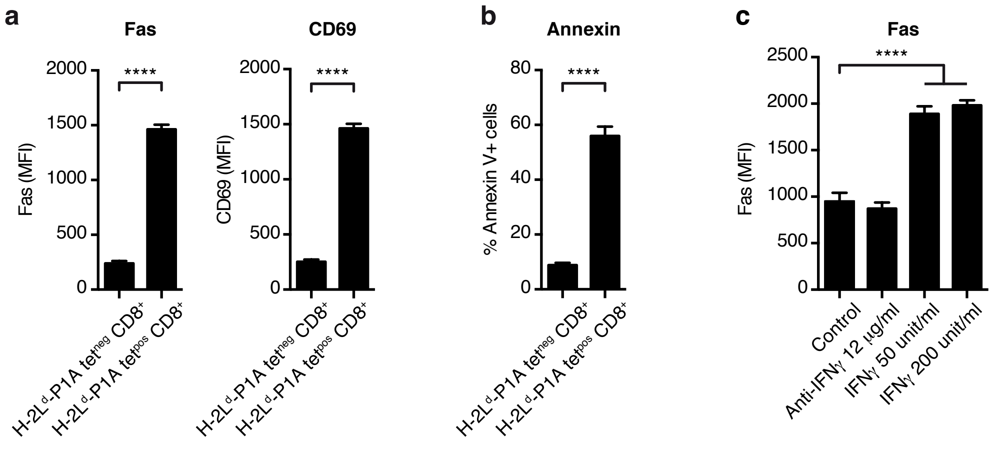

Supplementary Figure 10. Induction of Fas expression on CD8$^+$ T cells by antigenic activation

(**a**) Antigen-activated CD8$^+$ T cells upregulate Fas expression. Splenocytes isolated from induced-tumor bearing mice were incubated with irradiated L1210.P1A.B7-1 cells to activate the P1A-specific CD8$^+$ T cells. After 48 h, Fas expression was evaluated by FACS on P1A-specific or non-specific CD8$^+$ T cells identified by staining with the H-2L^d-P1A tetramer. CD69 expression was also evaluated to confirm T-cell activation. Fas expression was observed only on P1A-specific T cells, indicating that Fas is induced by T-cell activation in an antigen-specific manner. Three independent experiments, each performed in duplicate.

(**b**) Antigen-activated CD8$^+$ T cells are more sensitive to FasL mediated apoptosis. Splenocytes isolated and treated as described in (*a*) were incubated with 50 ng/ml recombinant FasL (a kind gift from Pascal Schneider, Lausanne, Switzerland) and apoptosis was evaluated by FACS staining for Annexin V after 24 hours.

(**c**) Interferon-gamma increases Fas expression on activated CD8$^+$ T cells. P1A-specific CD8$^+$ T cells isolated from TCRP1A-transgenic mice were activated *in vitro* by co-incubation with L1210.P1A.B7-1. After 4 days, they were purified with Lymphoprep and incubated with IFNγ or anti-IFNγ antibody as indicated. Twenty-four hours later, Fas expression was evaluated by FACS. Three independent experiments, each in duplicate. Results are expressed as mean + s.e.m. Unpaired t-test, two-tailed (*a*-*c*), ****$P<0.0001$.
